# 2067 Developing a cultural adaptation of a telephone genetic counseling intervention for Latina women at-risk of hereditary breast and ovarian cancer

**DOI:** 10.1017/cts.2018.249

**Published:** 2018-11-21

**Authors:** Alejandra Hurtado de Mendoza, Sara Gómez Trillos, Marc Schwartz, Beth Peshkin, Heidi E. Hamilton, Katie Fiallos, Filipa C. Lynce, Susan Vadaparampil, Vanessa B. Sheppard, Lucile Adams-Campbell, Andrea Otero, Kristi Graves

**Affiliations:** 1 Georgetown University – Howard Universities; 2 Virginia Commonwealth University; 3 Georgetown University; 4 Department of Genetic Counseling, Instituto de Medicina Oncológica y Molecular de Asturias (IMOMA), Oviedo, Spain

## Abstract

OBJECTIVES/SPECIFIC AIMS: The overall goal of this project is to enhance the use of GCRA in Latina breast cancer survivors at high risk of hereditary breast and ovarian cancer to reduce disparities in GCRA uptake. The aims of the study are to (1) develop a cultural adaptation of an evidence-based TGC intervention that consists of phone genetic counseling and a booklet, (2) evaluate the impact of TGC Versus Usual Care, and (3) explore the communication patterns in TGC and genetic counseling sessions with an interpreter. METHODS/STUDY POPULATION: We are conducting a 2-phase, mixed methods study. In Phase I we will develop a cultural adaption of an evidence-based intervention (TGC) for high-risk Latina breast cancer survivors using the Learner Verification and Revision Framework (n=15). In Phase II we will use a cluster randomized design with four community sites randomized to Spanish TGC (n=2 sites) or usual care (n=2 sites) (n=60; 15 per site). The primary outcome is genetic counseling uptake. Among women who receive genetic counseling either through TGC (n~30) or with an interpreter (n~15), we will assess counseling quality by reviewing 20 randomly selected audiotaped sessions (10 TGC; 10 interpreters). We will evaluate women’s HBOC knowledge and satisfaction with counseling. Communication processes and outcomes will be assessed using gold standard RIAS quantitative coding system and qualitative discourse analysis. RESULTS/ANTICIPATED RESULTS: We elicited input from transdisciplinary team members to develop an initial adaptation of a TGC print booklet and intervention protocol for use with high-risk Latina breast cancer survivors with limited English proficiency. The booklet contains low-literacy information about HBOC, risk factors, pros and cons of testing, and management strategies. Based on these materials and prior work, we anticipate TGC will consist of one 1 hour or less TGC session by phone. Participants interested in pursuing testing will receive a saliva kit and will participate in a second TGC session (30 min) to discuss test results and management options. DISCUSSION/SIGNIFICANCE OF IMPACT: Given access barriers and the shortage of Spanish-speaking genetic counselors, adapting and translating TGC intervention is a promising strategy that could reduce disparities by broadening the reach and accessibility to genetic counseling while enhancing the quality of the service for Latinas with limited English proficiency.

